# A targeted methicillin-resistant Staphylococcus aureus (MRSA) control program did not affect total nosocomial Staphylococcus aureus (SA) bloodstream infections (BSI) despite reducing MRSA BSI

**DOI:** 10.1186/1753-6561-5-S6-O5

**Published:** 2011-06-29

**Authors:** B Amri, A Vasudevan, J Li, LY Hsu, D Fisher, PA Tambyah

**Affiliations:** 1 National University of Singapore, Singapore, Singapore

## Introduction / objectives

Background: Intense efforts at reducing MRSA infections have been used in Singapore hospitals from 2007 with active surveillance, intense hand hygiene campaigns, public displays of surveillance results and audio and visual reminders.

Aim: We evaluated the impact of this campaign on overall Staphylococcus aureus (SA) bloodstream infection rates.

## Methods

Method: All patients with a positive blood culture for SA from Oct 2007 to Feb 2011 were prospectively studied. Infections were classified as community acquired (CA), healthcare acquired (HCA) or nosocomial (NA) according to the US CDC. Rates were calculated per 1000 patient days.

## Results

Results: There were a total of 554 SA bloodstream infections during the study period, 115 were CA, 265 HCA and 174 NA of these, 220 were MRSA (10 CA. 111 HCA and 99 NA) and 334 were methicillin susceptible (MSSA) (103 CA, 155 HCA and 76 NA). The trends were analysed using regression models. Despite a decrease in overall SA BSI from 0.62 ± 0.20 to 0.44 ± 0.13 per 1000 pt-days (rate -0.119, p=0.023), there was no significant change in nosocomial SA BSI (from 0.21 ± 0.08 to 0.15 ± 0.10, rate 0.043, p=0.16). This is because the decline (rate 0.0037, P= 0.0001) in nosocomial MRSA BSI from 0.16 ± 0.06 to 0.05 ± 0.09 per 1000 pt days was offset by a rise in nosocomial MSSA infections from 0.06 ± 0.05 to 0.10 ± 0.17 per 1000 pt days (increase rate 0.0013 P= 0.061).

## Conclusion

Conclusion: Our targeted MRSA control program reduced nosocomial MRSA BSI, however replacement by nosocomial MSSA attenuated the overall impact on BSI. A comprehensive approach is needed to reduce all hospital acquired infections.

## Disclosure of interest

B. Amri: None declared, A. Vasudevan: None declared, J. Li: None declared, L. Y. Hsu: None declared, D. Fisher: None declared, P. Tambyah Grant/Research support from Adamas, Baxter, Merlion, Consultant for Astra Zeneca, Novartis, Wyeth, Pfizer.

